# The Accessory Helix of Complexin Stabilizes a Partially Unzippered State of the SNARE Complex and Mediates the Complexin Clamping Function *In Vivo*

**DOI:** 10.1523/ENEURO.0526-20.2021

**Published:** 2021-04-05

**Authors:** Joshua Brady, Alexander Vasin, Maria Bykhovskaia

**Affiliations:** 1 Ophthalmology, Visual and Anatomical Sciences Department, Wayne State University School of Medicine, Detroit, MI 48202; 2 Neurology Department, Wayne State University School of Medicine, Detroit, MI 48202

**Keywords:** botulinum toxin, lipid bilayer, mEPSP, molecular dynamics, spontaneous transmission, synaptic vesicle

## Abstract

Spontaneous synaptic transmission is regulated by the protein complexin (Cpx). Cpx binds the SNARE complex, a coil-coiled four-helical bundle that mediates the attachment of a synaptic vesicle (SV) to the presynaptic membrane (PM). Cpx is thought to clamp spontaneous fusion events by stabilizing a partially unraveled state of the SNARE bundle; however, the molecular detail of this mechanism is still debated. We combined electrophysiology, molecular modeling, and site-directed mutagenesis in *Drosophila* to develop and validate the atomic model of the Cpx-mediated clamped state of the SNARE complex. We took advantage of botulinum neurotoxins (BoNTs) B and G, which cleave the SNARE protein synaptobrevin (Syb) at different sites. Monitoring synaptic depression on BoNT loading revealed that the clamped state of the SNARE complex has two or three unraveled helical turns of Syb. Site-directed mutagenesis showed that the Cpx clamping function is predominantly maintained by its accessory helix (AH), while molecular modeling suggested that the Cpx AH interacts with the unraveled C terminus of Syb and the SV lipid bilayer. The developed molecular model was employed to design new Cpx poor-clamp and super-clamp mutations and to tested the predictions *in silico* employing molecular dynamics simulations. Subsequently, we generated *Drosophila* lines harboring these mutations and confirmed the poor-clamp and super-clamp phenotypes *in vivo.* Altogether, these results validate the atomic model of the Cpx-mediated fusion clamp, wherein the Cpx AH inserts between the SNARE bundle and the SV lipid bilayer, simultaneously binding the unraveled C terminus of Syb and preventing full SNARE assembly.

## Significance Statement

Spontaneous release of neuronal transmitters is an important component of synaptic transmission, which controls neuronal development, homeostasis, and synaptic plasticity. The synaptic protein complexin (Cpx) regulates synaptic transmission by clamping spontaneous release. We developed and validated an atomic model of the Cpx-mediated clamping mechanism, which can be employed as a tool for generating predictions for site-directed mutagenesis and selectively manipulating spontaneous synaptic transmission.

## Introduction

Neuronal transmitters are packed in synaptic vesicles (SVs) and released by fusion of SVs with the presynaptic membrane (PM). The attachment of an SV to the PM is mediated by the SNARE complex (Südhof and Rothman, 2009; Südhof**,** 2013), a coil-coiled four-stranded helical bundle, which consists of an SV protein synaptobrevin (Syb) or v-SNARE, and PM-associated proteins, syntaxin (Syx) and SNAP25 or t-SNARE. The assembly of the SNARE bundle enables overcoming the electrostatic and hydration repulsion between the SV and PM lipid bilayers (Rizo and Xu**,** 2015). The rapid synchronous fusion of SVs with the PM is evoked by an influx of Ca^2+^ ions into the nerve terminal, and a protein Synaptotagmin 1 acts as a Ca^2+^ sensor (Chapman**,** 2008).

Fusion events can also occur in the absence of action potentials, in a spontaneous mode. Genetic, biochemical, and physiological studies have identified a cytosolic protein complexin (Cpx) as a molecular player that inhibits spontaneous fusion but promotes the evoked Ca^2+^-dependent release (Mohrmann et al., 2015; Trimbuch and Rosenmund, 2016). Cpx attaches to the SNARE complex forming a five-helical bundle (Chen et al., 2002). Cpx deletion produces a drastic increase in spontaneous fusion events (Huntwork and Littleton, 2007), suggesting that the energetic barrier for SV fusion is reduced (Giraudo et al., 2006). This function of Cpx as a fusion clamp is well established in invertebrates (Hobson et al., 2011; Martin et al., 2011; Jorquera et al., 2012; Wragg et al., 2013), but is more controversial in mammalian synapses (Trimbuch and Rosenmund, 2016). However, it was demonstrated that Cpx inhibits spontaneous activity at cortical neuronal cultures (Yang et al., 2013), at mammalian calyx of Held (Chang et al., 2015), and at ribbon synapses (Vaithianathan et al., 2013, 2015). Furthermore, promoting Cpx action by either genetic overexpression or supplementation inhibited exocytosis in neurosecretory cells (Itakura et al., 1999; Abderrahmani et al., 2004; Liu et al., 2007). Altogether a large body of literature shows that Cpx clamps spontaneous fusion both *in vitro* and *in vivo* (Mohrmann et al., 2015), although this Cpx function *in vivo* appears to be synapse specific and is more prominent in invertebrates.

Several important features of the clamping mechanism have been established: (1) different domains of Cpx control evoked and spontaneous transmission, and these two Cpx functions are not correlated (Xue et al., 2007; Cho et al., 2014); (2) Cpx clamping function is regulated by its accessory helix (AH; Kaeser-Woo et al., 2012; Cho et al., 2014; Vasin et al., 2016) and by its C-terminal domain (Kaeser-Woo et al., 2012); (3) lipid binding is important for the Cpx clamping function (Wragg et al., 2013); (4) single point mutations in the Cpx AH selectively alter the spontaneous release component (Cho et al., 2014; Vasin et al., 2016); and (5) replacing the Cpx AH by a non-native helix restores the clamping function (Radoff et al., 2014). Notably, the latter two findings appear to contradict each other, raising the question of the mechanism by which the Cpx AH controls spontaneous transmission.

Although multiple studies suggest that Cpx interferes with zippering of v-SNARE onto t-SNARE (Giraudo et al., 2006, 2009; Krishnakumar et al., 2011, 2015; Kümmel et al., 2011; Li et al., 2011; Bykhovskaia et al., 2013; Vasin et al., 2016; Zdanowicz et al., 2017), the exact structure of the partially unraveled SNARE bundle in its clamped state is still debated. It was initially proposed that v-SNARE is unraveled radically, up to the middle of the four-helical bundle (Giraudo et al., 2009; Krishnakumar et al., 2011; Kümmel et al., 2011; Li et al., 2011). However, it was also shown that the electrostatic repulsion between the PM and an SV decays rapidly with distancing (Bykhovskaia et al., 2013; Fortoul et al., 2015) and therefore it is unlikely to drive the separation between the bilayers required for such radical SNARE unzippering. Other studies (Bykhovskaia et al., 2013; Fortoul et al., 2015, 2018; Vasin et al., 2016) suggested that a more likely scenario is that only two or three membrane-proximal helical turns (layers 7–9) of v-SNARE are separated from t-SNARE. Botulinum neurotoxins (BoNTs) represent an advantageous tool to discriminate between these different scenarios, since BoNTs cleave the SNARE proteins at distinct peptide bonds, and the cleavage depends on the BoNT serotype (Montecucco and Schiavo, 1993; Link et al., 1994). Here, we combined BoNT loading, molecular modeling of protein-lipid complexes, and Cpx mutagenesis to build and validate a model of the Cpx-mediated fusion clamp.

## Materials and Methods

### *Drosophila* stocks and genetics

All *Drosophila melanogaster* fly stocks were cultured on standard medium at a temperature of 22°C. The following stocks were used: *cpx* null mutant *cpx*−/− [*cpx^SH1^*; Huntwork and Littleton (2007); obtained from the lab of J. T. Littleton]; wild-type (WT) Canton-S and C155 *elev*-Gal4 (Bloomington Drosophila Stock Center, Indiana University). Epoch Life Science was used for site directed mutagenesis of Cpx (Isoform Cpx 7A; Buhl et al., 2013). The PCR products were subcloned into a pValum construct, which enabled the use of the Gal4/UAS system for gene expression (Brand and Perrimon, 1993). The constructs were injected by BestGene for a targeted third chromosome insertion into yv;;attp2 site. Homozygote third chromosome UAS lines were recombined into the *cpx*−/− null background (*cpx^SH^*^1^; Huntwork and Littleton, 2007). The C155 *elav*-Gal4 driver was used to pan-neuronally express the mutated transgenes.

### Electrophysiology

The third instar larvae were dissected in HL3 solution composed of the following: 70 mm NaCl, 5 mm KCl, 20 mm MgCl_2_, 10 mm NaHCO_3_, 5 mm trehalose, 115 mm sucrose, 2.5 mm HEPES-HCl, 2.5 mm HEPES-NaOH, and 1 mm CaCl_2_ (pH 7.2–7.4). EPSPs and miniature EPSPs (mEPSPs) were recorded from neuromuscular junctions (NMJs) using the focal macropatch technique (Vasin and Bykhovskaia, 2017). To record selectively from 1b type boutons, we used DIC optics and 60× water immersion objective (Olympus 0.95 NA) with a 2-mm working distance. Recordings of excitatory postsynaptic potentials (EPSPs) and miniature EPSPs (mEPSPs) were performed with macropatch electrodes with tip diameters of 5 μm and 1-MΩ seal resistances and digitized using a Digitdata A/D board and Axoscope software (Molecular Devices). Nerve stimulation was performed with Master-8 pulse stimulator (AMPI). The recordings were analyzed using Quantan software (Bykhovskaia, 2008).

### Loading nerve terminals with BoNTs

BoNT serotypes BoNT/B or BoNT/G (1 mg/ml, Metabiologics Inc) mixed (1:1) with fluorescent dye rhodamine B isothiocyanate (Sigma-Aldrich) conjugated to a 10-kDa dextran (RITC-dextran, concentration between 40 and 200 μm) were loaded into nerve terminals through the cut axon. We have adopted the protocol developed for loading Ca^2+^ indicators in *Drosophila* terminals (Rossano and Macleod, 2007). The second abdominal muscle segment was used in these experiments to minimize the potential variability. The axon was cut to a length of ∼0.5 mm from the site of the muscle innervation and inserted into the suction electrode. The suction electrodes were fire polished and had the opening diameter of 2–3 μm, so that the axon would fit tightly and there would be no solution exchange between the bath and the suction electrode. The suction electrode was filled with the mixture of BoNT and RITC, positioned near the cut axon, and the axon was rapidly suctioned into the electrode. Subsequently, the recording electrode was rapidly positioned over a 1b bouton as close to the suction electrode as possible to minimize the BoNT diffusion time Evoked transmission was elicited via the same suction electrode.

### Immunohistochemistry

Dissected larvae were fixed for 45 min in HL3 saline containing 4% formaldehyde. Following washing in PBST (0.1% Triton X-100 containing 1× PBS solution), larvae were preincubated in the blocking solution containing 2% normal goat serum, 2% bovine serum albumin, and 0.05% sodium azide for 1 h. Primary antibody was applied overnight at 4°C. The secondary antibody was applied for 4–6 h at the room temperature.

For Cpx immunostaining, *Drosophila* anti-Cpx antibody (1:200), a generous gift from J. T. Littleton (Huntwork and Littleton, 2007; Cho et al., 2014) and a secondary goat anti-rabbit polyclonal Cy3 IgG antibody (1:200, Novus Biologicals) were used. For the horseradish peroxidase (HRP) labeling, the preparations were incubated with anti-HRP conjugated to Alexa Fluor 488 (1:250, Jackson ImmunoResearch). To detect BoNT, we used a polyclonal monovalent antibody specific to BoNT/B (1:100, Metabiologics) and a secondary mouse anti-rabbit IgG antibody (1:100, Life Technologies).

Fluorescence was visualized and imaged using Velocity software (Improvision) on a laser-based confocal microscope (PerkinElmer) with an ORCA-ER CCD camera (Hamamatsu) using an oil-immersion 50×/0.9 objective (Olympus).

### Molecular dynamics

The molecular systems were constructed using Visual Molecular Dynamics Software (VMD, Theoretical and Computational Biophysics Group, NIH Center for Macromolecular Modeling and Bioinformatics, at the Beckman Institute, University of Illinois at Urbana-Champaign). All the simulations were performed in a water/ion environment with explicit waters. Potassium and chloride ions were added to neutralize the systems and to yield a 150 mm concentration of KCl. Water boxes with added ions were constructed using VMD. The phosphatidylcholine (POPC) lipid bilayers mimicking an SV were generated using VMD. The initial structure of anionic lipid bilayer containing phosphatidylserine (POPS) and phosphatidylinositol 4,5-bisphosphate (PIP_2_), POPC:POPS:PIP_2_ (75:20:5) mimicking the PM (Alwarawrah and Wereszczynski, 2017) was kindly provided by J. Wereszczynski (Illinois Institute of Technology). In all the systems, the lipid bilayers were positioned in the *xy***-**plane.

The MD simulations were performed employing CHARMM36 force field (Vanommeslaeghe et al., 2010) modified to include the parameters for PIP_2_ as described previously (Alwarawrah and Wereszczynski, 2017). The simulations were performed with periodic boundary conditions and Ewald electrostatics in the NPT ensemble at 310K. The heating (20 ps) and equilibration (100 ns) phases were performed employing NAMD (Phillips et al., 2005) Scalable Molecular Dynamics (Theoretical and Computational Biophysics Group, NIH Center for Macromolecular Modeling and Bioinformatics, at the Beckman Institute, University of Illinois at Urbana-Champaign) at XSEDE (Extreme Science and Engineering Discovery Environment) Stampede cluster (TACC). The NAMD simulations were performed with a flexible cell and with a time-step of 1.5 fs, employing Langevin thermostat and Berendsen barostat. Production runs were performed at Anton2 supercomputer (Shaw, 2014; Shaw et al., 2009) with Desmond software through the MMBioS (National Center for Multiscale Modeling of Biological Systems, Pittsburgh Supercomputing Center and D.E. Shaw Research Institute). All the Anton2 simulations were performed in a semi-isotropic regime, with a time-step of 2.5 fs, and employing the multigrator (Lippert et al., 2013) to maintain constant temperature and pressure. The trajectory analysis was performed employing VMD and Vega ZZ (Drug Design Laboratory) software. All the parameters along all the trajectories were computed with a time step of 2.4 ns.

### Statistical analysis

One-way ANOVA followed by the Tukey’s test was employed to evaluate statistical significance.

## Results

**Figure 1.**
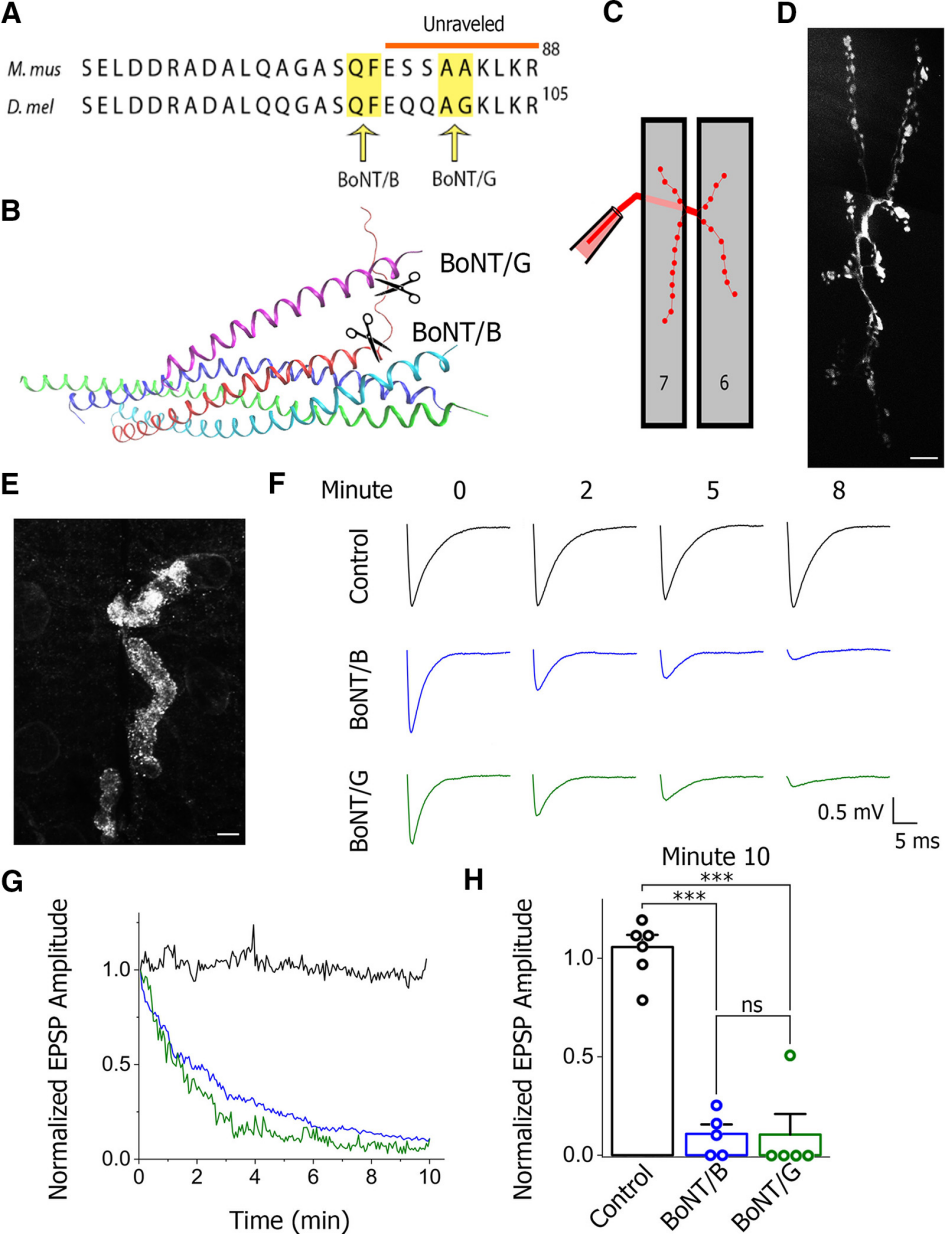
Loading BoNT in the nerve terminals. ***A***, Cleavage sites for BoNT/B and BoNT/G in the mammalian Syb and *Drosophila* n-syb. ***B***, The sites of BoNT/B and BoNT/G cleavage shown at the model of the clamped state of the SNARE-Cpx complex. Note that the BoNT/G cleavage site is situated within the unraveled C terminus of Syb, while the BoNT/B cleavage site is situated within the zippered Syb region. ***C***, The diagram illustrating the protocol of the BoNT loading through the cut axon innervating the muscles 6 and 7 of the *Drosophila* NMJ. ***D***, The RITC dye mixed with BoNT diffused through the entire NMJ on BoNT loading. ***E***, Immunostaining shows the presence of BoNT/B in the nerve terminals in the end of the experiment. ***F–H*,** Evoked transmission is rapidly reduced on BoNT loading. Examples of recorded EPSPs (***F***) illustrate that EPSP amplitude diminishes within minutes in BoNT/B and BoNT/G loaded preparations (blue and green), but not in control baseline recordings (black). The kinetics of the EPSP decay (***G***) shows a rapid decrease for both BoNT/B (blue) and BoNT/G (green) versus control. The data are normalized by the EPSP amplitude of the first response. Each data point represents an average of five experiments. In the end of the recording (***H***) the EPSP amplitude in BoNT loaded preparations is significantly reduced compared with the baseline control; ***p < 0.001. ns - not significant.

We took advantage of botulinum toxins BoNT/B (Schiavo et al., 1992) and BoNT/G (Yamasaki et al., 1994), which cleave Syb at two different sites (Fig. 1*A*). Transgenic expression of BoNTs in *Drosophila* demonstrated that both serotypes cleave the *Drosophila* neuronal Syb (n-syb; Backhaus et al., 2016). Our initial goal was to test the “late clamp” model (Bykhovskaia et al., 2013; Vasin et al., 2016), which proposed that the clamped state of the SNARE-Cpx complex involves the unraveled layers 7–9 of the SNARE bundle (Fig. 1*A*,*B*). As such, this model predicts that n-syb will be accessible for the cleavage by BoNT/G but not by BoNT/B (Fig. 1*B*) when the SNARE complex is clamped. Conversely, a more radically unzippered n-syb would be accessible to cleavage by BoNT/B.

BoNT was loaded through the cut axon (Fig. 1*C*; Rossano and Macleod, 2007). To monitor loading, we added RITC to the solution and ensured that the dye reached the nerve terminals (Fig. 1*D*). To ensure the presence of BoNT in the terminals, at the end of the recordings we fixed the preparations and performed immunostaining for BoNT/B (Fig. 1*E*). In addition, we assessed BoNT activity by monitoring evoked release on the nerve stimulation, since at these conditions the pool of docked SVs is rapidly depleted, and therefore the EPSP amplitude should decay rapidly (Hua et al., 1998). To assess whether this is the case, we stimulated the nerve continuously at a 3-Hz frequency while loading either BoNT/B or BoNT/G. For both BoNT serotypes, we observed rapid decay in EPSP amplitudes (Fig. 1*F*), and within 10 min the evoked activity was almost entirely eliminated (Fig. 1*F*,*G*).

**Figure 2.**
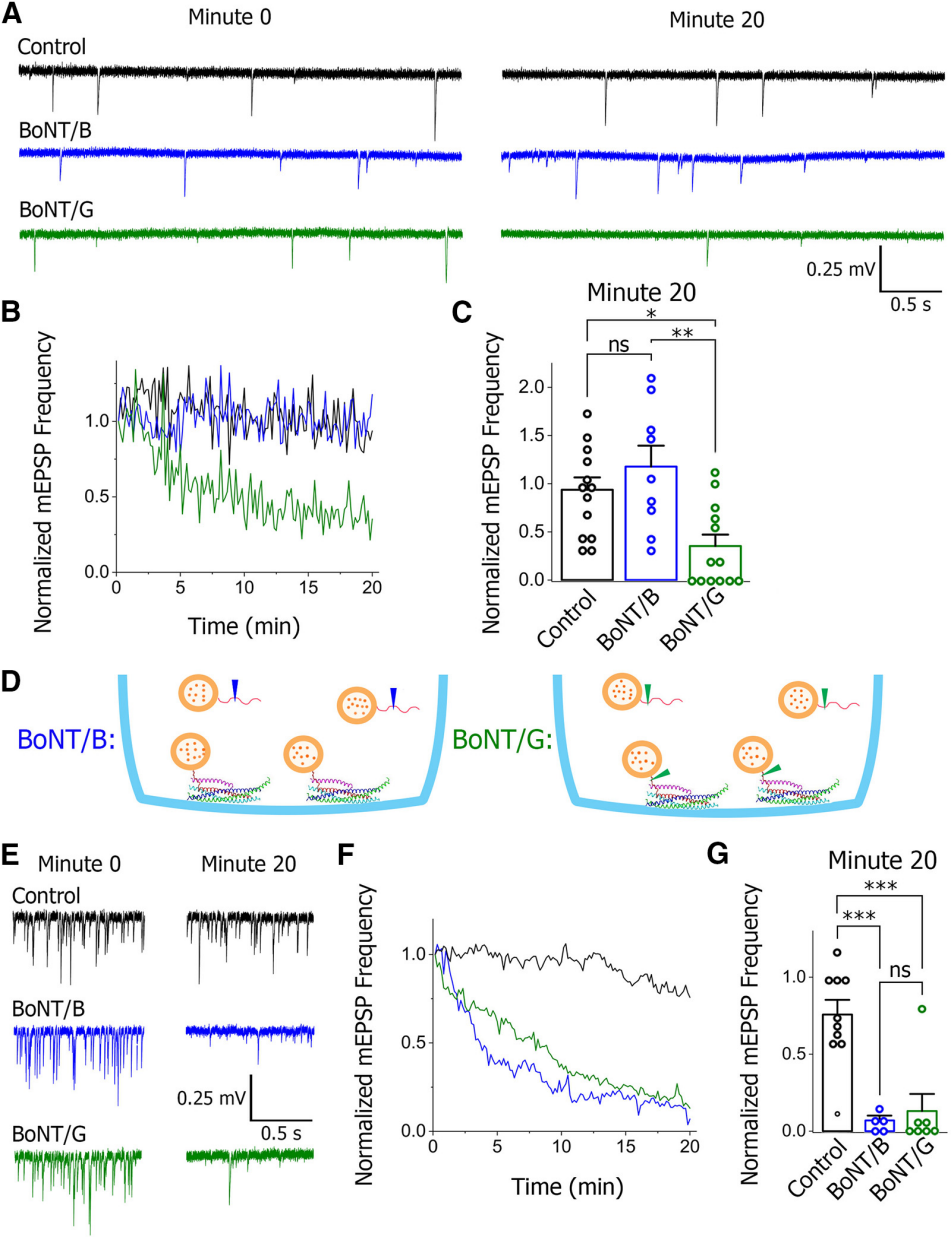
Spontaneous transmission decays on BoNT/G but not on BoNT/B loading, and the differential effect of BoNTs is abolished in *cpx−/−* NMJs. ***A–C***, In WT preparations, spontaneous transmission significantly decays within 20 min BoNT/G but not BoNT/B loading. Representative traces (***A***) that illustrate that after 20 min of BoNT loading spontaneous transmission is reduced in the BoNT/G loaded but not in the BoNT/B loaded NMJ. The mEPSP frequency is reduced over time (***B***) in BoNT/G loaded but not in BoNT/B loaded preparations. The mEPSP frequency is binned over 10-s intervals and normalized by the first bin in each experiment. In the end of 20-min loading, the mEPSP frequency is significantly reduced compared with the control baseline in BoNT/G but not in BoNT/B loaded preparations; *p < 0.05, **p < 0.01, ***p < 0.001. ***D***, The diagram illustrating that BoNT/G but not BoNT/B would cleave Syb of the clamped SNARE-Cpx complexes of docked SVs. ***E–G***, The *cpx*−/− preparations show rapid decay in the mEPSP frequency on BoNT loading because of high rates of spontaneous transmission. However, the differential effect of BoNT/B versus BoNT/G is abolished. ***p < 0.001. Representative traces (***E***) illustrate the reduction in spontaneous transmission after 20 min BoNT loading. The mEPSP frequency is rapidly reduced over time (***F***) in BoNT/G and BoNT/B preparations. In the end of 20-min loading, the mEPSP frequency is significantly reduced compared with the control baseline in both BoNT/G and BoNT/B loaded preparations. ns - not significant.

Next, we loaded the preparations with BoNT/B or BoNT/G and monitored the decay in spontaneous synaptic activity. BoNT/G loading produced a rapid decay in spontaneous transmission (Fig. 2*A–C*, green). In contrast, there was essentially no decay after BoNT/B loading within 20 min (Fig. 2*A–C*, blue). These results suggest that n-syb is accessible for BoNT/G cleavage but to a lesser extent for BoNT/B cleavage. This is consistent with the model wherein BoNT/B does not cleave the clamped SNARE complexes of docked SVs (Fig. 2*D*, blue), while BoNT/G does (Fig. 2*D*, green). Indeed, the rate of spontaneous transmission is low (∼0.7–0.8 Hz per bouton; Vasin et al., 2016) and the number of SVs docked to the PM is large (∼1000 per bouton; Meinertzhagen et al., 1998; Sabeva et al., 2017), and therefore docked SVs can maintain spontaneous transmission for 20 min or longer, accounting for steady spontaneous transmission in BoNT/B loaded preparations.

Since the model proposes that the clamped state of the SNARE complex with partially unraveled Syb is stabilized by Cpx, it could be expected that the differential effects of BoNT/G and BoNT/B serotypes would be abolished for *cpx*−/− NMJs. To test whether this is the case, we recorded spontaneous transmission from *cpx*−/− NMJs on BoNT/G versus BoNT/B loading. We found that the differential effect of the BoNT/B and BoNT/G serotypes was completely abolished in *cpx*−/− preparations (Fig. 2*E–G*). Indeed, spontaneous transmission at *cpx*−/− NMJs decayed rapidly on loading either BoNT/G or BoNT/B. This could be expected because of high rates of spontaneous transmission in *cpx*−/− NMJs (∼80 Hz; Vasin et al., 2016), which should rapidly deplete the pool of docked SVs.

**Figure 3.**
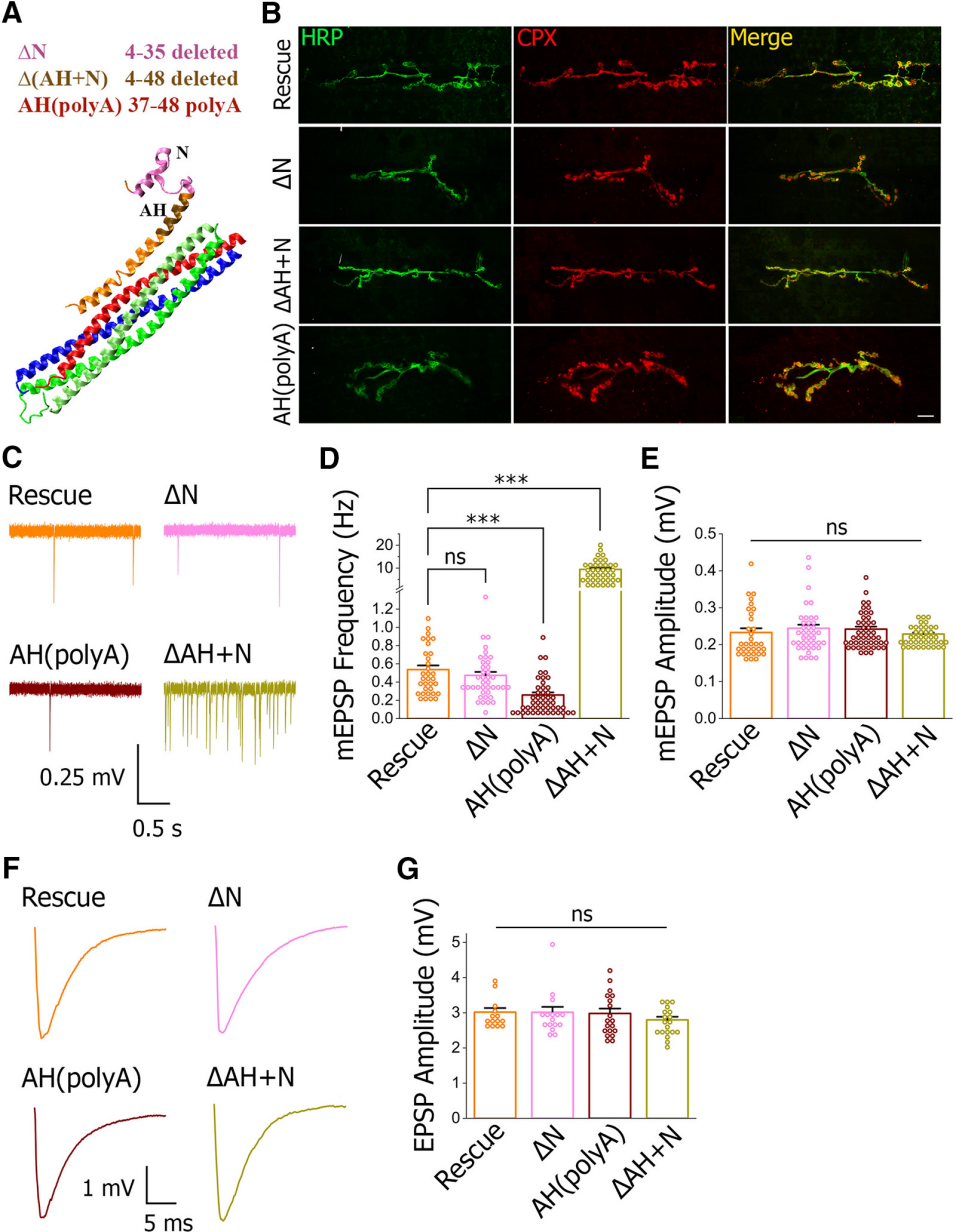
Cpx AH has a pivotal role in the regulation of spontaneous transmission. ***A***, The diagram showing the design of the mutations in relevance to the structure of the SNARE-Cpx complex. Red: Syb; blue: Syx; green: SNAP25; orange: Cpx. ***B***, All the mutants show a normal Cpx expression pattern, with Cpx localized to the nerve terminals. Images show the double labeling for the neuronal marker HRP and immunostaining for Cpx, which are co-localized. Scale bar: 10 μm. ***C***, ***D***, Cpx AH but not N-terminal domain regulates the frequency of spontaneous transmission. Representative traces (***C***) and mEPSP frequencies (***D***) show that spontaneous transmission is not altered in the *cpx^ΔN^* mutant (pink), is significantly reduced in the *cpx^AH(poly A)^* mutant (maroon), and is drastically enhanced in the *cpx^Δ(N+AH)^* mutant (ochre); ***p < 0.001. ***E***, The mutations do not alter mEPSP amplitudes. ***F***, ***G***, Evoked transmission is not altered in either of the mutants. Representative traces (***F***) and EPSP amplitudes (***G***) do not show any significant alterations in either of the mutants. ns - not significant.

We next employed Cpx mutagenesis to delineate the role of the Cpx AH in the clamping mechanism. The following mutants have been generated (Fig. 3*A*): (1) deletion of the Cpx AH and N terminus (*cpx^Δ(AH+N)^*, residues 4–48); (2) deletion of the N terminus only (*cpx^ΔN^*, residues 4–35); and (3) replacement of the Cpx AH by the poly A sequence (*cpx^AH(poly A)^*, residues 37–48 being replaced). All the mutated Cpx forms were expressed in the *cpx*−/− background. We have ascertained that the mutated proteins are properly expressed in the nerve terminals (Fig. 3*B*).

We found that the mEPSP frequency in the *cpx^Δ(AH+N)^* mutant was ∼17-fold higher than in the control rescue line (Fig. 3*C–E*, ochre vs orange), although it was not as high as in *cpx*−/− (∼80 Hz; Vasin et al., 2016). Interestingly, the *cpx^ΔN^* mutant (Fig. 3*C*,*D*, pink) had an unaltered mEPSP frequency compared with the control, showing that the spontaneous activity was inhibited by the Cpx AH region. Notably, replacing the native AH sequence with the poly A sequence in the *cpx^AH(poly A)^* mutant decreased the mEPSP frequency significantly below the control level (Fig. 3*C*,*D*, maroon vs orange). None of the mutants showed any significant alterations in either mEPSP amplitude (Fig. 3*E*) or evoked transmission (Fig. 3*F*,*G*) compared with the rescue control.

These results clearly show that the Cpx AH is required for the Cpx clamping function while the Cpx N-terminal domain has no role in it. Further, these results show that the structure of the Cpx AH is fine-tuned for incomplete clamping, since its replacement with a poly A sequence actually enhances the Cpx clamping function. How could the poly A sequence be more functional than the native sequence? The simplest explanation is that the Cpx AH is inserted between the SNARE bundle and an SV thus creating an extra barrier between the PM and an SV. Since the poly A sequence is hydrophobic, it would likely have a high affinity to an SV and could therefore create a more reliable barrier than the native Cpx AH. To explore this possibility, we performed molecular modeling of the SNARE-Cpx complex between lipid bilayers mimicking the PM and an SV.

**Figure 4.**
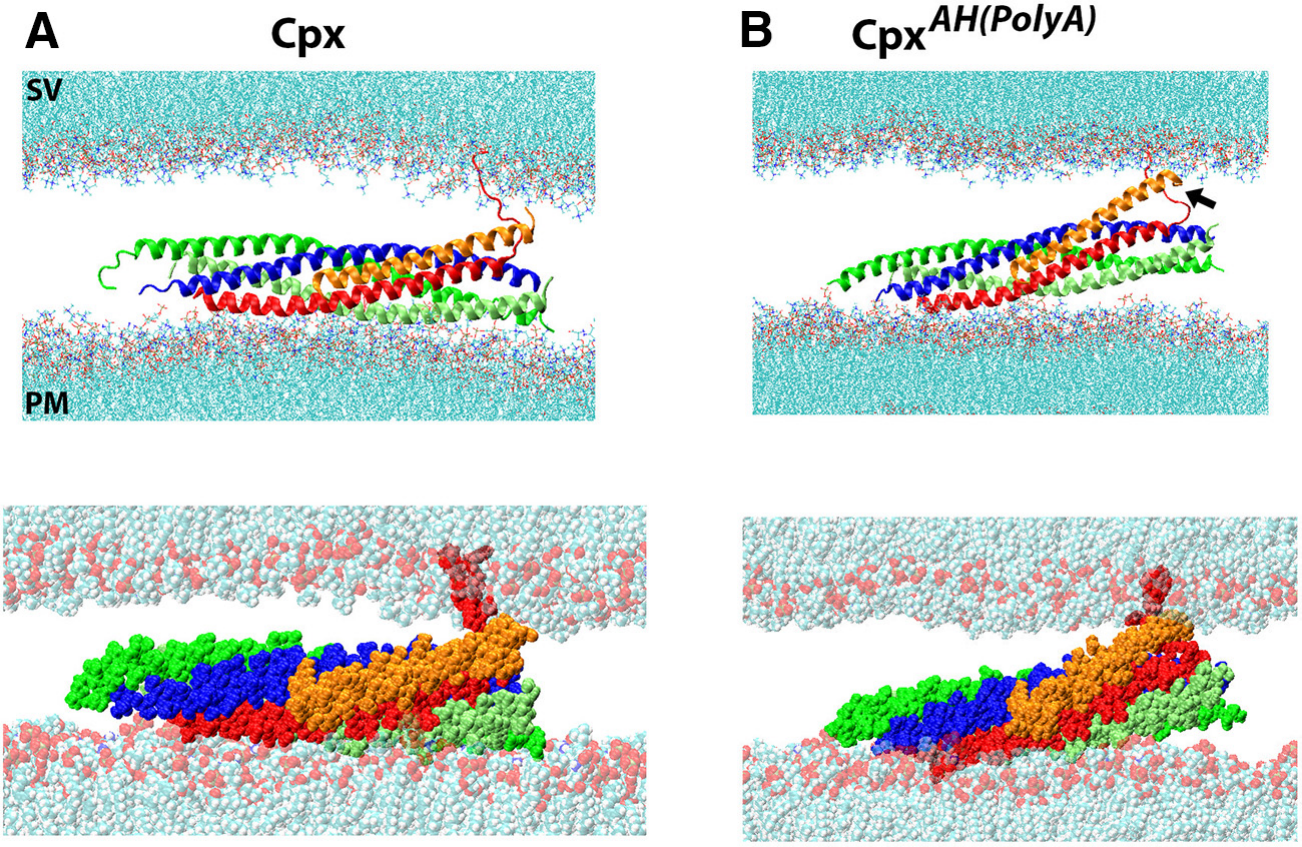
The all-atom model of the clamped SNARE-Cpx complex between lipid bilayers mimicking the PM and an SV. Cartoon (top) and VdW (bottom) representations of the SNARE-Cpx complex are shown. ***A***, Native Cpx (orange) stabilizes the unraveled C terminus of Syb (red) and also interacts with the SV bilayer. ***B***, The Cpx^AH(poly^ *^A^*^)^ mutant interacts tightly with the SV bilayer (arrow) enhancing the barrier between the SV and the PM.

The structure of the *Drosophila* SNARE-Cpx bundle with partially unraveled n-syb (Vasin et al., 2016) was positioned between the bilayers. The distance between the bilayers was adjusted to 3 nm since at this distance the bilayers do not interfere with the SNARE-Cpx bundle, the C-terminal residues of n-syb become embedded in the SV bilayer, and the electrostatic repulsion between the bilayers is not prominent (Bykhovskaia et al., 2013; Fortoul et al., 2015). We then performed 300 ns of MD simulations for this molecular system. The system remained stable, the Cpx AH remained positioned between the SNARE bundle and the SV bilayer, the unraveled C terminus of n-syb (layers 7–9) remained separated from the bundle, and the C-terminal residues of n-syb remained embedded in the SV bilayer (Fig. 4*A*). We then replaced the Cpx AH (residues 37–48) with the poly A sequence and repeated the simulations. Notably, we found that at the end of the trajectory the mutated Cpx AH interacted with the bilayer mimicking an SV more tightly, reinforcing the barrier between the bilayers (Fig. 4*B*, arrow).

**Figure 5.**
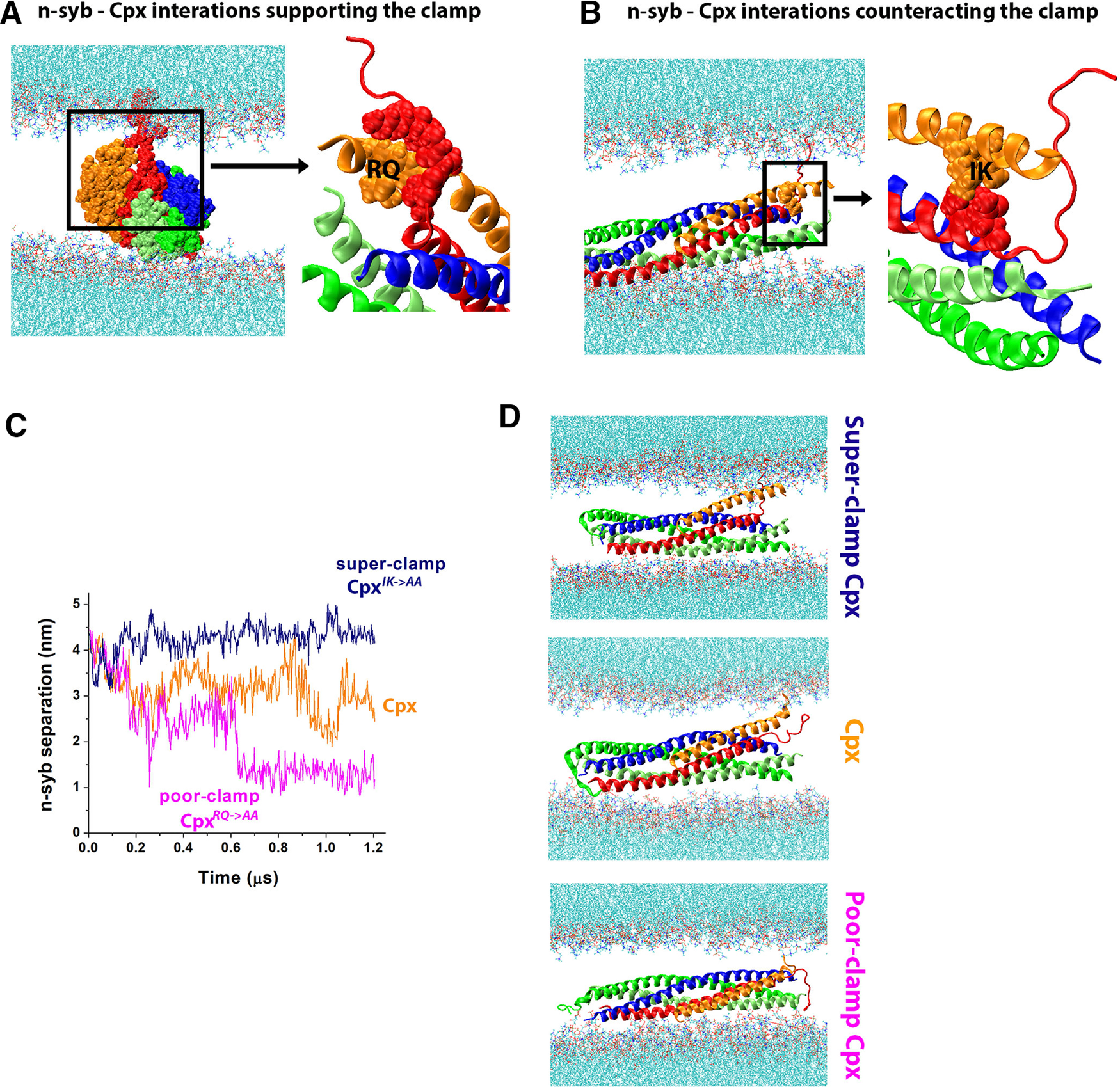
The design and *in silico* testing of the Cpx poor-clamp and super-clamp mutants. ***A***, The poor-clamp mutant is designed to disrupt the RQ motif within the Cpx AH, which maintains the interactions between the Cpx AH and the unraveled n-syb terminus. ***B***, The super-clamp mutant is designed to disrupt the IK motif maintaining the interactions between the Cpx AH and the zippered part of the SNARE bundle. ***C***, MD simulations of the native and mutated complexes show that the super-clamp mutant maintains the separation between the unraveled n-syb C terminus and the SNARE bundle, while the poor-clamp mutant does not. The graph shows the distance between C_α_ atoms of the C-terminal residues of n-syb and Syx over the length of the 1.2-μs MD trajectory. Trajectory points are separated by 240 ps. ***D***, Structures of the mutated and native Cpx complexes in the end of respective MD trajectories. Note the unraveled C terminus of n-syb and the Cpx AH tightly interacting with the SV bilayer in the super-clamp mutant. In contrast, in the poor-clamp mutant, the C terminus of n-syb separated from the SV bilayer and started forming contacts with t-SNARE.

We next asked whether this model of the fusion clamp (Fig. 4*A*) has predictive power and could guide us in the Cpx mutagenesis. To test this, we designed two point mutations in the Cpx AH: poor-clamp and super-clamp. To design the poor-clamp mutation, we examined the atomic model of the clamped SNARE-Cpx complex to identify the Cpx residues which stabilize the unraveled state of n-syb. We found that these interactions (Fig. 5*A*) predominantly involve the residues R43 and Q44 of the Cpx AH (RQ motif; Fig. 5*A*, boxed region), which are conserved between mammalian and *Drosophila* Cpx forms (R37 and Q38 in the mammalian Cpx1). Therefore, the model predicts that *cpx^RQ->AA^* mutation would produce the poor-clamp phenotype.

The model also predicts that the super-clamp phenotype can be created by loosening the attachment of the Cpx AH to the SNARE bundle. Such mutation would fortify the clamp by separating the Cpx AH from the bundle, enhancing the interactions of the Cpx AH with the SV lipid bilayer, and thus augmenting the barrier between the SNARE bundle and the SV, similar to the *cpx^AH(poly A)^* mutant (Fig. 4*B*). The examination of the atomic model revealed tight links between the SNARE bundle and the IK motif of Cpx (Fig. 5*B*, residues I47 and K48, homologous to L41 and R42 in mammalian Cpx1). Therefore, it could be predicted that mutating this motif would enhance the clamping function of Cpx.

First, we tested these predictions *in silico*. The molecular systems (Fig. 4*A*) with the mutated Cpx forms (Cpx*^RQ->AA^*, poor-clamp and Cpx*^IK->AA^*, super-clamp) were generated, and prolonged MD simulations (for 1.2 μs) for each system were performed. To monitor the separation of n-syb from the SNARE bundle, we measured the distance between Cα atoms of the C-terminal residues of n-syb an Syx along each trajectory. In this *in silico* molecular system, zippering of n-syb onto t-SNARE would serve as a measure for the ability of the SNARE complex to fully assemble and drive the SV-PM fusion. We found that the molecular system with the Cpx*^IK->AA^* mutant had n-syb separated from the t-SNARE bundle for the entire length of the trajectory (Fig. 5*C*, navy), and this clamped state was supported by the Cpx AH, which interacted with the unraveled C terminus of n-syb and with the SV bilayer (Fig. 5*D*, top). In contrast, the system containing the Cpx*^RQ->AA^* mutant did not stabilize. In this system, the unraveled C terminus of n-syb separated from the bilayer mimicking an SV and attached to the SNARE bundle (Fig. 5*C*, magenta, *D*, bottom), although full SNARE zippering was not observed at our time-scale (1.2 μs). Finally, the system containing native Cpx showed an intermediate behavior, with the C terminus of n-syb remaining partially unraveled at the end of the trajectory (Fig. 5*C*, orange, *D*, middle). These results demonstrate *in silico* that the Cpx*^IK->AA^* mutant would likely stabilize the clamped state of the SNARE complex, producing the super-clamp phenotype, while the Cpx*^RQ->AA^* mutant would not be able to maintain the clamp and would likely show the poor-clamp phenotype.

**Figure 6.**
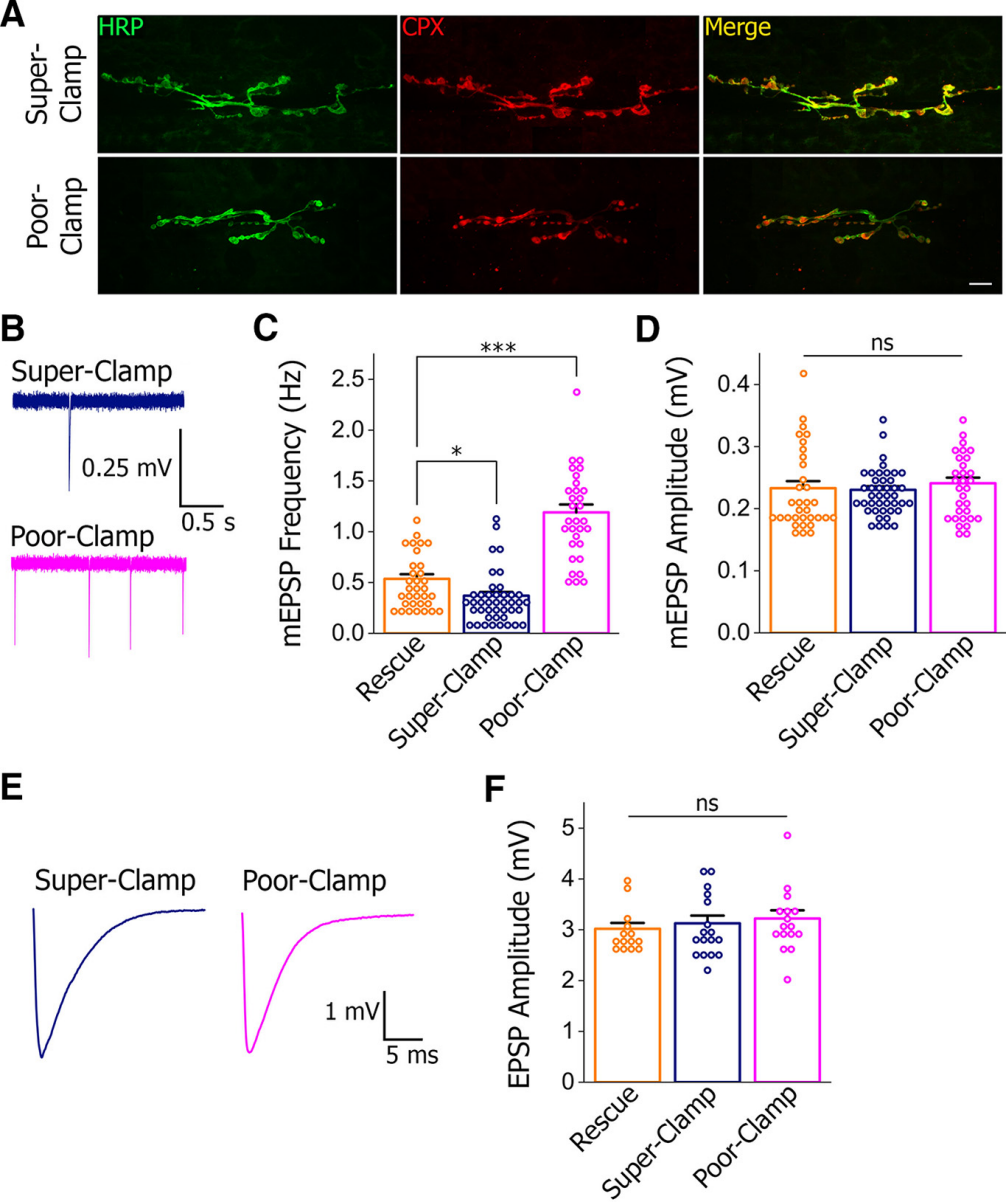
Testing the super-clamp and poor-clamp mutations *in vivo.* ***A***, Both mutants show a normal Cpx expression pattern, with Cpx co-localized with the neuronal marker HRP. Scale bar: 10 μm. ***B***, ***C***, Recording traces (***B***) and EPSP frequencies (***C***) show that spontaneous transmission is significantly increased in the poor-clamp mutant and significantly decreased in the super-clamp mutant; *p < 0.05, ***p < 0.001. ***D***, mEPSP amplitudes are not affected by the mutations. ***E***, ***F***, The mutations do not affect evoked transmission, as evident from representative EPSP traces (***E***) and average EPSP amplitudes (***F***). ns - not significant.

We then tested these predictions *in vivo*. The mutated *cpx* genes *cpx^IK->AA^* and *cpx^RQ->AA^* were expressed in the *cpx*−/− background. Both mutated forms of Cpx were properly expressed in the nerve terminals (Fig. 6*A*). We then assayed spontaneous transmission in both lines. We found that the mEPSP frequency was more than doubled in the *cpx^RQ->AA^* mutant compared with the control rescue line (Fig. 6*B*,*C*). Thus, as predicted by the model, the *cpx^RQ->AA^* mutant showed the poor-clamp phenotype, although quantitatively the effect of the RQ->AA mutation in the Cpx AH was approximately 8-fold below the effect of the Cpx AH deletion (Fig. 3). In contrast, the mEPSP frequency was significantly reduced in the *cpx^IK->AA^* mutant showing, as predicted, the super-clamp phenotype. Neither mEPSP amplitude (Fig. 6*D*) nor evoked transmission (Fig. 6*E*) was affected in either of the mutants.

Altogether, our results support a model of the clamped state of the SNARE complex with only two or three C-terminal layers of Syb being unraveled. Our results also suggest that this clamped state of the SNARE complex is stabilized by the Cpx AH being inserted between the SNARE bundle and the SV lipid bilayer, while also interacting with the partially unraveled C terminus of Syb. Finally, we show that this molecular model enables designing poor-clamp and super-clamp mutations and thus selectively manipulating Cpx clamping function and spontaneous synaptic transmission.

## Discussion

Cpx is a potent regulator of synaptic transmission, and its function has been studied extensively (Mohrmann et al., 2015; Trimbuch and Rosenmund, 2016). Several molecular models have been developed to address the mechanism by which Cpx inhibits spontaneous transmission (Giraudo et al., 2009; Kümmel et al., 2011; Li et al., 2011; Bykhovskaia et al., 2013). Most of the models agree that Cpx stabilizes a partially unraveled state of the SNARE complex, thus preventing full SNARE zippering and spontaneous fusion events. However, the specific molecular detail of this mechanism is still debated. Further, it is largely agreed that the Cpx AH has a pivotal role in the clamping mechanism; however, it is not yet clear how specifically it mediates the Cpx clumping function.

One of the earliest models (Giraudo et al., 2009) suggested that the Cpx AH intercalates between the unraveled Syb and the t-SNARE bundle. Subsequently, this model was modified to incorporate several SNARE complexes cross-linked by Cpx molecules (Kümmel et al., 2011; Krishnakumar et al., 2015). A more recent study proposed that Cpx competes with Syb for binding to t-SNARE and thus inhibits SNARE assembly (Zdanowicz et al., 2017). The studies cited above suggested that Cpx radically interferes with the SNARE assembly, preventing the major part of Syb from zippering onto the t-SNARE bundle. An alternative late clamp model (Bykhovskaia et al., 2013; Vasin et al., 2016) proposed that Cpx only interferes with the final stages of SNARE zippering, inhibiting two or three SV-proximal helical turns of Syb from zippering onto t-SNARE.

Interestingly, an *in vivo* study in *Caenorhabditis elegans* demonstrated that the replacement of the Cpx AH with a non-native helix (multiple repeats of the EAAK motif) did not change spontaneous transmission (Radoff et al., 2014). This study suggested that it is the helical structure of the Cpx AH and not its specific sequence which is critical for the Cpx clamping function. However, this finding appears to be in contradiction with several *in vivo* studies in mouse (Yang et al., 2010) and *Drosophila* (Cho et al., 2014; Vasin et al., 2016) which showed that point mutations within the Cpx AH sequence can produce poor-clamp or super-clam phenotypes.

We took advantage of the *Drosophila* preparation, which shows a very strong and robust effect of Cpx on spontaneous transmission (Huntwork and Littleton, 2007; Jorquera et al., 2012). The first question we asked was: how radically is the SNARE complex unzippered in its clamped state? To elucidate this question, we took advantage of clostridial neurotoxins (Niemann et al., 1994; Pellegrini et al., 1995; Xu et al., 1998; Hua and Charlton, 1999), a tool which has been broadly used to understand how SNARE proteins control exocytosis. We used BoNT serotypes B and G, which cleave Syb (n-syb in *Drosophila*) at two different sites: near layer 7 (BoNT/G) and near layer 6 (BoNT/B). We reasoned that if Syb is unzippered by only two or three helical turns in its clamped state, as suggested by the late clamp model (Bykhovskaia et al., 2013), then we would observe a faster depression of spontaneous release on BoNT/G loading since BoNT/G (but not BoNT/B) would cleave docked SVs attached to the PM by partially unraveled SNARE complexes. In contrast, if Syb is separated from t-SNARE more radically, as suggested by other models (Giraudo et al., 2009; Kümmel et al., 2011), then both BoNT serotypes would cleave partially zippered SNARE complexes and, respectively, produce similar decays in spontaneous transmission. Our results supported the first scenario, with BoNT/G producing a significantly stronger reduction in spontaneous transmission than BoNT/B.

In line with these findings, our Cpx mutagenesis experiments coupled with molecular modeling showed that the region of the Cpx AH (11 residues forming three helical turns) is critical for the Cpx clamping function, and that this Cpx region would likely interact with the unraveled region of Syb. Indeed, removal of the Cpx AH produced an ∼17-fold increase in the mEPSP frequency. In should be noted, however, that this increase in spontaneous synaptic activity did not approach in magnitude the effect of the Cpx deletion (Huntwork and Littleton, 2007; Jorquera et al., 2012), which showed over 100 increase in the mEPSP frequency when measured by the same technique as in the present study (Vasin et al., 2016). Thus, although Cpx AH represents an important structural element for clamping spontaneous fusion, it does not completely account for this Cpx action. What other Cpx motifs may contribute to fusion clamping? Our study showed that this Cpx function does not depend on its N-terminal domain, since its deletion did not alter spontaneous transmission. However, an earlier study at the mouse cultured neurons demonstrated that the Cpx clamping function depends on its C-terminal domain (Kaeser-Woo et al., 2012). Further investigation is needed to understand how the Cpx AH and its C-terminal domain interact in controlling spontaneous transmission.

Notably, we also found that replacing this Cpx region with a poly A sequence significantly reduces the mEPSP frequency compared with the control rescue line. This finding agrees with the results obtained in *C. elegans*, which showed that replacing the Cpx AH with a non-native helix rescues the Cpx function (Radoff et al., 2014). Furthermore, our finding shows that a non-native helix, such as the poly A sequence, can clamp spontaneous transmission even better than the native Cpx AH. This result suggests that the Cpx sequence is fine-tuned to regulate the rate of spontaneous transmission but not designed to maximally block it. This is not surprising, since the spontaneous release is an important component of synaptic transmission, required for neuronal development and several forms of plasticity (Kavalali, 2015; Andreae and Burrone, 2018).

To understand how the Cpx AH clamps fusion, we have developed a molecular model of the SNARE-Cpx complex between lipid bilayers mimicking an SV and the PM. Our modeling suggested that the Cpx AH could clamp fusion by creating a barrier between the SNARE bundle and the SV bilayer while simultaneously interacting with the unraveled C terminus of Syb. These three-partied interactions, involving the partially unraveled C terminus of Syb, the Cpx AH, and the SV bilayer would stabilize the clamped state of the SNARE complex. In this model, a more hydrophobic Cpx AH (such as the poly A sequence) could produce a better clamp because of more tight interactions with the SV bilayer.

We then combined computations and experiments to test whether this model could guide us in targeted mutagenesis. To produce the poor-clamp phenotype, we mutated two residues within the Cpx AH (Cpx*^RQ->AA^*) which were predicted to bind the unraveled Syb terminus. Testing this mutation *in silico* suggested that indeed, the poor- clamp mutation would accelerate SNARE zippering. We next generated the *Drosophila* line harboring the poor-clamp mutation and demonstrated that the spontaneous transmission in this mutant was increased more than twice compared with the control rescue. One could argue that the mutation could reduce the Cpx expression levels, however, this explanation is highly unlikely. Indeed, earlier studies (Cho et al., 2014; Vasin et al., 2016) showed that mutations within the Cpx AH do not affect either Cpx expression or delivery to the nerve terminals, and the immunolabeling data obtained in the present study argues that this not the case for the poor-clamp Cpx*^RQ->AA^* mutant. Instead, our results suggest that the poor-clamp phenotype is produced by the compromised interactions of the Cpx AH with the n-syb unraveled terminus, as predicted by the model. Notably, the quantitative effect of the poor-clamp mutation (∼2-fold increase) is much smaller than the effect of the Cpx AH deletion (∼17-fold). This result is in line with our model, which suggests that the clamping mechanism enabled by the Cpx AH includes two components: (1) binding the partially unraveled C terminus of n-syb and preventing its full zippering; and (2) creating a barrier between the SV and the SNARE bundle and thus separating the SV from the PM. These two mechanisms are likely to act synergistically. Indeed, the attachment of the Cpx AH to an SV would promote the separation of the Cpx AH from the SNARE bundle and, respectively would stabilize the partially unzippered state of Syb. Since the Cpx*^RQ->AA^* mutant can only compromise the first but not the second mechanism, it is not surprising that the effect produced by this mutation is modest. The atomic model developed here enables systematic targeted manipulations by the two mechanisms and evaluating their quantitative impact. For example, the model predicts that the fusion clamp can be enhanced by fortifying the interactions of the Cpx AH with the SV bilayer by substituting the Cpx residues that face away from the SNARE bundle by more hydrophobic residues, such as Ala, Phe, Trp, or Leu. Furthermore, the model also predicts that the fusion clamp can be fortified by weakening the interactions between Cpx AH and the SNARE bundle.

To test the latter prediction, we designed the super-clamp mutation by disrupting the interactions of the Cpx AH with the zippered part of the SNARE bundle, thus promoting the interactions of the Cpx AH which mediate the fusion clamp. A similar strategy for designing super-clamp single point mutations in Cpx and n-syb was employed in an earlier study (Vasin et al., 2016). Testing the super-clamp mutation *in silico* showed that the mutation would slow down zippering of the SNARE complex, and the *Drosophila* line harboring the super-clamp mutation showed a significant and selective decrease in the spontaneous transmission.

These results promote mechanistic understanding of how Cpx interactions fine-tune spontaneous synaptic transmission and provide the strategy for selectively manipulating the spontaneous release component.

## Synthesis

Reviewing Editor: Kristen Harris, The University of Texas at Austin Decisions are customarily a result of the Reviewing Editor and the peer reviewers coming together and discussing their recommendations until a consensus is reached. When revisions are invited, a fact-based synthesis statement explaining their decision and outlining what is needed to prepare a revision will be listed below. The following reviewer(s) agreed to reveal their identity: Silvio Rizzoli.

Summary comments and items that should be addressed in the revised manuscript.

Introductory remarks:

The manuscript improves our knowledge on complexin function, especially on the potential role of complexin during synaptic transmission, which is an important advance. They use an elegant combination of imaging and electrophysiology in Drosophila, as well as molecular simulations, to suggest a detailed model of how complexin interacts with SNARE molecules. They start by relying on suggestions from the effects of botulinum neurotoxins B and G, which cleave VAMP (synaptobrevin) in different locations, and follow this up by site-directed mutagenesis of complexin, to suggest that the function of complexing in clamping the SNARE complex is mediated by its accessory helix. Molecular simulations then suggest that this helix interacts with the C-terminus of VAMP. Finally, they follow up on this suggestion and validate their model by introducing new mutations suggested by the simulations. The authors examine and test molecular models for how the fusion clamp Complexin (Cpx) prevents unregulated zippering of the neuronal SNARE complex to regulate spontaneous synaptic vesicle release rates. Drosophila null mutants of Cpx display a >40-fold enhancement in spontaneous mini release and a reduction in evoked release. The authors use this system, together with molecular modeling, to examine how Cpx’s clamping function might arise from its interactions with SNAREs and membrane lipids. The authors provide evidence that Cpx clamps the SNAREs when they are near fully zippered, in contrast to other findings that suggest Cpx clamps the SNAREs in a much looser state where more complete unzippering has been proposed. The evidence for this is two-fold. First, they show Cpx clamped vesicles are protected from BoNT/B but not BoNT/G, suggesting SNAREs are likely be tightly zippered in the docked and primed state. Second, using a combination of molecular modeling and structure-function approaches, the authors argue Cpx simultaneously interacts with the SNARE complex and the synaptic vesicle membrane during its clamping state.

Main items that must be addressed:

1. All bar graphs should be presented as box plots or violin plots, which would indicate better the spread of the data.

2. Fix Figure 3 labeling. The polyA rescue (brown) in C is mislabeled with the greenish deltaAH+N rescue in panels D, E and G. Panel D, for example, shows the polyA mutant doesn’t rescue the mini defect, when in fact it should be the delta AH + N mutant that doesn’t rescue.

3. Tone down claim on point mutants and explain why null mutant has 80 Hz mini frequency rate and the poor clamp mutant has 1.2 Hz mini frequency -- one could actually argue this result disproves their model, though the poor clamp does have a 2-fold increase in minis over controls, so there is a small effect. They should acknowledge this in the discussion, so readers are not misled.

4. Further discussion of whether it is the repulsion from the SV membrane or the block in SNARE zippering that is the major point they want to make - confusion arises as to whether both things are equally important, or if the authors favor one of the mechanisms over the other.

5. It would have been nice to see quantitative westerns, but the reviewer did not think this lab is well equipped for that approach. Hence, it would be acceptable to include a statement in the discussion that the authors cannot rule out small changes in Cpx proteins levels could also contribute to the phenotypes.

In addition, the following detailed items should be addressed:

1. There are a couple of places where there are alternative explanations or caveats with the interpretation that would be worth the authors addressing (or at least noting) in their discussion.

a. The effects of the poor-clamp and super-clamp mutants are very mild on spontaneous release rates, so the authors should note there must be additional molecular effects that these mutants don’t recapitulate for Cpx’s clamping function. With the current discussion, a non-expert in the field would assume the authors’ model accounts for all the clamping function of Cpx, when in fact that is not the case. The poor-clamp mutant increases mini frequency to around ∼1.2 Hz compared to ∼0.5 Hz in controls. Cpx mutants have a mini frequency rate of ∼ 80 Hz, so the poor-clamp is strikingly different from the null. The mini rate in the null increases ∼160-fold, while the poor-clamp mutant only increases mini rates ∼2-fold. There is still > 95% of the Cpx null mini phenotype that is unaccounted for by this mutation (and likely by the model as well). We still have much to learn, and the authors should point out this key issue so naïve readers don’t overinterpret their results.

b. Figure 2e-g interpretation. The authors suggest the ability of BoNT/B to reduce spontaneous release rates in Cpx mutants is due to the fact that Cpx clamps the SNARE complex secondary to a partially unraveled Syb and this function is lost in Cpx mutants. The differential sensitivity of BoNT/B versus BoNT/G is very clear and argues for this interpretation in the wildtype recordings. In the Cpx mutants, the reason BoNT/B can now work to reduce mini rate is that the dramatic increase in Cpx mutants simply cycles more SVs through the exo-endocytotic cycle, where Syb now becomes susceptible to cleavage when it is no longer docked with the t-SNAREs. As such, the most obvious interpretation of this result is that the Cpx mutant just drives SV recycling so much you deplete docked SVs and then become sensitive to this cleavage site during SV cycling. It’s a nice result to show the differential sensitivity at different times of the SV cycle, but one could argue you would see the same thing if you increased SV cycling through any other mechanism with Cpx there or not (as the authors already report for evoked release in wildtype, that is sensitive to both toxins).

2. It is strange that the authors use alanine substitutions for their poor-clamp and super-clamp mutants since they show early on that a complete substitution of the AH with a poly alanine stretch rescues the null mutant phenotype just fine. The logic is unclear - why then pick a few of the sites to change to alanines that subsequently give opposite phenotypes. Since they work overall in the AH, why does only changing a subset not work? Would be worth addressing this more clearly in the discussion in terms of the polyA full rescue versus the point mutant alanine substitutions.

4. It would be nice to show western blots and quantification of the amount of Cpx expressed in the rescue mutants, given the extreme dosage sensitivity of synaptic transmission by Cpx reported in the field. If any of these mutants alter Cpx stability or levels, that could be a confound for the phenotypes. Given the most important of the mutants, the polyA substitution of the AH, rescues the phenotype, this concern applies more to the poor clamp and super-clamp mutations which only have mild effects on spontaneous release rates that could be contributed to changes in Cpx levels if either of the mutant proteins are less stable.

5. Some questions on the modeling could be cleared up with more discussion on the following points:

a. It seems the C-terminus of Syb is unconstrained other than weak interactions with the SV bilayer. The modeling of poor-clamp Cpx shows the C terminus of Syb is able to freely swing around to interact with t-SNAREs. In reality, the Syb C terminus is constrained by the transmembrane domain anchoring it in the SV bilayer. Any thoughts on how transmembrane anchoring of the SNAREs would alter the model?

b. Confusion about what the authors consider to be the key aspect of the modeling from the two scenarios at play: 1) Cpx’s accessory helix binding to the synaptic vesicle membrane and pushing the vesicle farther away to inhibit full SNARE zippering by acting as a boundary (Fig. 4B); or 2) preventing SNARE zippering by binding to Syb (Fig. 5B)?

If the principle clamping mechanism of Cpx is to hold SNARE layers 7-9 apart via its central helix, one might expect a stronger affinity of the accessory helix to the SV membrane to disturb Cpx function, not enhance it. They note that the polyA mutant of Cpx creates a stronger boundary between the SV and PM, but their modeling appears to show less Cpx interaction with the actual SNAREs, as if the polyA mutation is pulling Cpx away from its clamping job and is instead causing it to associate more with the SV. This could also: 1) loosen the interaction of the central helix to the SNAREs (increasing the probability of full zippering) or 2) draw the SV closer to the PM (increasing the probability of fusion). This doesn’t appear to be the case, but the text does not explain why this doesn’t occur. A bit more discussion on these two mechanisms and how they interact to control clamping would make it a bit more understandable to the reader.

## Author Response

Response to the review

1. All bar graphs should be presented as box plots or violin plots, which would indicate better

the spread of the data.

In the revised manuscript, we depicted all the data point in the graphs, to show the spread of the

data as well as the numbers of observations.

2. Fix Figure 3 labeling. The polyA rescue (brown) in C is mislabeled with the greenish

deltaAH+N rescue in panels D, E and G. Panel D, for example, shows the polyA mutant doesn’t

rescue the mini defect, when in fact it should be the delta AH + N mutant that doesn’t rescue.

Indeed, figure 3 was mislabeled in the original manuscript. We thank the reviewers for pointing

this out, and we have corrected figure 3 in the revised manuscript.

3. Tone down claim on point mutants and explain why null mutant has 80 Hz mini frequency rate

and the poor clamp mutant has 1.2 Hz mini frequency -- one could actually argue this result

disproves their model, though the poor clamp does have a 2-fold increase in minis over controls,

so there is a small effect. They should acknowledge this in the discussion, so readers are not

misled.

In the revised manuscript we explicitly stated that quantitatively the deletion of the Cpx AH

produces a much weaker affect than the Cpx deletion (P. 8), and also that the poor clamp

mutation in the Cpx AH produces a much weaker effect than the deletion of the Cpx AH (P. 11

and the end of P. 14). We explain in the revised manuscript (P.13) that our data suggest that

other Cpx motifs, besides its AH, contribute to the clamping function, and we speculate that the

contribution of the Cpx C-terminus is likely. Furthermore, we explain (P. 15) that our data

suggests that the Cpx clamping function is enabled by the synergism of two mechanisms: the

interactions of the Cpx AH with n-syb and its interactions with the SV lipid bilayer, which

promote the role of the Cpx AH as a barrier. We explain (P. 15) that the poor-clamp mutation

inhibits the former interactions but not the latter.

4. Further discussion of whether it is the repulsion from the SV membrane or the block in SNARE

zippering that is the major point they want to make - confusion arises as to whether both things

are equally important, or if the authors favor one of the mechanisms over the other.

We favor the hypothesis that the two mechanisms work synergistically, and we state this in the

revised Discussion (P. 14-15):

"Notably, the quantitative effect of the poor-clamp mutation (approximately two-fold increase) is

much smaller than the effect of the Cpx AH deletion (approximately 17 fold). This result is in

line with our model, which suggests that the clamping mechanism enabled by the Cpx AH

includes two components: 1) binding the partially unraveled C-terminus of n-syb and preventing

2

its full zippering; and 2) creating a barrier between the SV and the SNARE bundle and thus

separating the SV from the PM. These two mechanisms are likely to act synergistically. Since

the CpxRQ->AA mutant can only compromise the first but not the second mechanism, it is not

surprising that the effect produced by this mutation is modest. The atomic model developed here

enables systematic targeted manipulations by the two mechanisms and evaluating their

quantitative impact, which is the subject for the future studies."

We believe that further modeling and experimentation is needed to determine the specific

quantitative contribution for each mechanism and to determine whether their contributions are

additive, or whether they mutually reinforce each other, or whether they can compensate for each

other. This is the subject for further studies.

5. It would have been nice to see quantitative westerns, but the reviewer did not think this lab is

well equipped for that approach. Hence, it would be acceptable to include a statement in the

discussion that the authors cannot rule out small changes in Cpx proteins levels could also

contribute to the phenotypes.

We addressed this point in the revised Discussion (P. 14):

"One could argue that the mutation could reduce the Cpx expression levels, however, this

explanation is highly unlikely. Indeed, earlier studies (Cho et al 2014, Vasin et al 2016) showed

that mutations within the Cpx AH do not affect either Cpx expression or delivery to the nerve

terminals, and the immunolabeling data obtained in the present study argues that this not the case

for the poor-clamp CpxRQ->AA mutant."

In addition, the following detailed items should be addressed:

1. There are a couple of places where there are alternative explanations or caveats with the

interpretation that would be worth the authors addressing (or at least noting) in their discussion.

a. The effects of the poor-clamp and super-clamp mutants are very mild on spontaneous release

rates, so the authors should note there must be additional molecular effects that these mutants

don’t recapitulate for Cpx’s clamping function. With the current discussion, a non-expert in the

field would assume the authors’ model accounts for all the clamping function of Cpx, when in

fact that is not the case. The poor-clamp mutant increases mini frequency to around ∼1.2 Hz

compared to ∼0.5 Hz in controls. Cpx mutants have a mini frequency rate of ∼ 80 Hz, so the

poor-clamp is strikingly different from the null. The mini rate in the null increases ∼160-fold,

while the poor-clamp mutant only increases mini rates ∼2-fold. There is still > 95% of the Cpx

null mini phenotype that is unaccounted for by this mutation (and likely by the model as well).

We still have much to learn, and the authors should point out this key issue so naïve readers

don’t overinterpret their results.

3

As explained in #2, we stated in the revised manuscript that the effect of the Cpx AH deletion

does not match quantitatively the effect of the deletion of the entire Cpx (P. 8):

"We found that the mEPSP frequency in the cpxΔ(AH+N) mutant was approximately 17 fold higher

than in the control rescue line (Fig. 3 C-E, ochre versus orange), although it was not as high as in

cpx-/-

(approximately 80 Hz, (Vasin et al 2016))."

We discuss this in P. 13:

"Indeed, removal of the Cpx AH produced an approximately 17-fold increase in the mEPSP

frequency. In should be noted, however, that this increase in spontaneous synaptic activity did

not approach in magnitude the effect of the Cpx deletion (Huntwork & Littleton 2007, Jorquera

et al 2012), which showed over 100 increase in the mEPSP frequency when measured by the

same technique as in the present study (Vasin et al 2016). Thus, although Cpx AH represents an

important structural element for clamping spontaneous fusion, it does not completely account for

this Cpx action. What other Cpx motifs may contribute to fusion clamping? Our study showed

that this Cpx function does not depend on its N-terminus domain, since its deletion did not alter

spontaneous transmission. However, an earlier study at the mouse cultured neurons demonstrated

that the Cpx clamping function depends on its C-terminal domain (Kaeser-Woo et al 2012).

Further investigation is needed to understand how the Cpx AH and its C-terminal domain

interact in controlling spontaneous transmission. "

We also state that the effect of the poor-clamp mutation does not match quantitatively the effect

of the deletion of the Cpx AH (P. 11):

"Thus, as predicted by the model, the cpx

RQ->AA mutant showed the poor-clamp phenotype,

although quantitatively the effect of the RQ->AA mutation in the Cpx AH was approximately

eight-fold below the effect of the Cpx AH deletion (Fig. 3)."

We then discuss this in P.14-15:

"Notably, the quantitative effect of the poor-clamp mutation (approximately two-fold increase) is

much smaller than the effect of the Cpx AH deletion (approximately 17 fold). This result is in

line with our model, which suggests that the clamping mechanism enabled by the Cpx AH

includes two components: 1) binding the partially unraveled C-terminus of n-syb and preventing

its full zippering; and 2) creating a barrier between the SV and the SNARE bundle and thus

separating the SV from the PM. These two mechanisms are likely to act synergistically. Indeed,

the attachment of the Cpx AH to an SV would promote the separation of the Cpx AH from the

SNARE bundle and, respectively would stabilize the partially unzippered state of Syb. Since the

CpxRQ->AA mutant can only compromise the first but not the second mechanism, it is not

surprising that the effect produced by this mutation is modest."

b. Figure 2e-g interpretation. The authors suggest the ability of BoNT/B to reduce spontaneous

release rates in Cpx mutants is due to the fact that Cpx clamps the SNARE complex secondary to

a partially unraveled Syb and this function is lost in Cpx mutants. The differential sensitivity of

4

BoNT/B versus BoNT/G is very clear and argues for this interpretation in the wildtype

recordings. In the Cpx mutants, the reason BoNT/B can now work to reduce mini rate is that the

dramatic increase in Cpx mutants simply cycles more SVs through the exo-endocytotic cycle,

where Syb now becomes susceptible to cleavage when it is no longer docked with the t-SNAREs.

As such, the most obvious interpretation of this result is that the Cpx mutant just drives SV

recycling so much you deplete docked SVs and then become sensitive to this cleavage site during

SV cycling. It’s a nice result to show the differential sensitivity at different times of the SV cycle,

but one could argue you would see the same thing if you increased SV cycling through any other

mechanism with Cpx there or not (as the authors already report for evoked release in wildtype,

that is sensitive to both toxins).

The issue of SV recycling is still debated, and we are not convinced that the reviewers’

interpretation is correct. Indeed, electron microscopy studies (Jorquera et al., J Neurosi., 2012;

Sabeva et al., J. Neurosci. 2017) unambiguously demonstrated that Cpx deletion does not reduce

the number of docked SVs, and these finding raise the question of how SVs recycle in cpx-/-

boutons and what mechanisms enable accommodating the massive spontaneous release observed

in cpx-/-. For example, one of the EM studies (Sabeva et al., J. Neurosci. 2017) suggested that

the SV recycling may be modified in cpx-/- boutons, and that active zone recycling may be

preferred. In line with this hypothesis, our data (Figures 1 and 2) showed that the depletion for

the evoked release in WT boutons occurs much faster than the depletion for spontaneous release

in cpx-/- . This finding cannot be explained by the difference in the amount of release. Indeed,

we estimate that at 3 Hz stimulation frequency used in the present study the macropatch

electrode would detect approximately 250 quanta per minute (this estimate is based on quantal

content m=14; Vasin et al., Biophys. J. 2016). The spontaneous release in cpx-/- boutons would

amount to approximately 4800 quanta per minute, which is 19 times higher. Notably, our results

showed that the evoked stimulation in the presence of toxins produced almost complete depletion

in WT boutons within five minutes (Fig. 1 G). In contrast, cpx-/- synapses show a similar

depression level only after 20 min of the toxin exposure (Fig. 2 F). This result points to the

difference in the recycling pathways for evoked and spontaneous release components. In

addition, these results suggest, in agreement with EM data, that sufficient pool for docked SVs is

available for spontaneous release in cpx-/- boutons. Further experimentation is needed to

understand how the recycling pathway in cpx-/- boutons is modified. We agree with the reviewer

that BoNT loading could be a powerful tool to study SV recycling. However, although this is an

exciting prospect, such experiments are out of the scope of the present study.

2. It is strange that the authors use alanine substitutions for their poor-clamp and super-clamp

mutants since they show early on that a complete substitution of the AH with a poly alanine

stretch rescues the null mutant phenotype just fine. The logic is unclear - why then pick a few of

the sites to change to alanines that subsequently give opposite phenotypes. Since they work

overall in the AH, why does only changing a subset not work? Would be worth addressing this

more clearly in the discussion in terms of the polyA full rescue versus the point mutant alanine

substitutions.

5

Alanine substitutions are used broadly for targeted mutagenesis, and this is because alanine does

not possess a side chain which could have an action of its own, complicating the interpretation of

the results. Besides alanine, the only amino acid which would not impose this problem, is

glycine. However, glycine substitution may produce a helix break, which again, may have

consequences beyond the single point mutation. Therefore, in our view alanine substitutions are

optimal for targeted mutagenesis. For the poor-clamp and super-clamp mutations, we substituted

the Cpx residues which would interact with the SNARE bundle and not with an SV bilayer, and

therefore, we would not expect that these alanines would change Cpx interactions with lipid

bilayers. Our model makes a testable prediction that the super-clamp phenotype in the cpx

AH-

>polyA

mutant results from the substitution of the subset of residues facing away from the SNARE

bundle. We state this in the revised manuscript (P. 15):

"For example, the model predicts that the fusion clamp can be enhanced by fortifying the

interactions of the Cpx AH with the SV bilayer by substituting the Cpx residues that face away

from the SNARE bundle by more hydrophobic residues, such as Ala, Phe, Trp, or Leu."

4. It would be nice to show western blots and quantification of the amount of Cpx expressed in

the rescue mutants, given the extreme dosage sensitivity of synaptic transmission by Cpx

reported in the field. If any of these mutants alter Cpx stability or levels, that could be a

confound for the phenotypes. Given the most important of the mutants, the polyA substitution of

the AH, rescues the phenotype, this concern applies more to the poor clamp and super-clamp

mutations which only have mild effects on spontaneous release rates that could be contributed to

changes in Cpx levels if either of the mutant proteins are less stable.

As outlined in #5, we have addressed this concern in the revised Discussion (P. 14).

5. Some questions on the modeling could be cleared up with more discussion on the following

points:

a. It seems the C-terminus of Syb is unconstrained other than weak interactions with the SV

bilayer. The modeling of poor-clamp Cpx shows the C terminus of Syb is able to freely swing

around to interact with t-SNAREs. In reality, the Syb C terminus is constrained by the

transmembrane domain anchoring it in the SV bilayer. Any thoughts on how transmembrane

anchoring of the SNAREs would alter the model?

The model is designed to evaluate the propensity of n-syb to zipper onto the core SNARE bundle

for different complexes, and this designed is based on the notion that the SNARE complexes

with intact transmembrane domains would bring an SV and the PM together for those complexes

which zipper more readily. Of course, it would be ideal to model the SNARE proteins with intact

transmembrane domains and to show which of them can trigger the SV-PM attachment.

However, such design would require molecular systems of considerably larger sizes, and

simulating multiple runs for large such systems at the microsecond scale is not realistic with the

computational resources presently available. Therefore, we had to design our simulations with

reduced systems, and transmembrane domains were not included. However, our design implies

6

that n-syb zippering in the presence of transmembrane domains would drive the fusion. This is

stated in the revised manuscript (P. 10 ):

"In this in silico molecular system, zippering of n-syb onto t-SNARE would serve as a measure

for the ability of the SNARE complex to fully assemble and drive the SV-PM fusion. "

b. Confusion about what the authors consider to be the key aspect of the modeling from the two

scenarios at play: 1) Cpx’s accessory helix binding to the synaptic vesicle membrane and

pushing the vesicle farther away to inhibit full SNARE zippering by acting as a boundary (Fig.

4B); or 2) preventing SNARE zippering by binding to Syb (Fig. 5B)?

If the principle clamping mechanism of Cpx is to hold SNARE layers 7-9 apart via its central

helix, one might expect a stronger affinity of the accessory helix to the SV membrane to disturb

Cpx function, not enhance it. They note that the polyA mutant of Cpx creates a stronger

boundary between the SV and PM, but their modeling appears to show less Cpx interaction with

the actual SNAREs, as if the polyA mutation is pulling Cpx away from its clamping job and is

instead causing it to associate more with the SV. This could also: 1) loosen the interaction of the

central helix to the SNAREs (increasing the probability of full zippering) or 2) draw the SV

closer to the PM (increasing the probability of fusion). This doesn’t appear to be the case, but

the text does not explain why this doesn’t occur. A bit more discussion on these two mechanisms

and how they interact to control clamping would make it a bit more understandable to the

reader.

We believe that the two mechanisms act synergistically, and this is stated in the revised

manuscript (P. 14-15) :

"This result is in line with our model, which suggests that the clamping mechanism enabled by

the Cpx AH includes two components: 1) binding the partially unraveled C-terminus of n-syb

and preventing its full zippering; and 2) creating a barrier between the SV and the SNARE

bundle and thus separating the SV from the PM. These two mechanisms are likely to act

synergistically."

The first principle of the clamping mechanism proposed in our manuscript is that the accessory

helix (not central helix, as the reviewers stated) holds the layers 7-9 apart. This is clearly shown

in Fig. 1 B and Fig. 3 A. As the reviewer correctly noted, we believe that the polyA superclamping mutations are pulling Cpx away from the SNARE complex and to the SV. However,

this does not mean that these mutations are "pulling Cpx away from its clamping job", as the

reviewer states. Instead, we believe that the Cpx "clamping job" is to separate the SV from tSNARE, and this "clamping job" is enhanced when the Cpx AH attaches to the SV and pulls

away from t-SNARE. We added this explanation to the revised Discussion (P. 15):

"Indeed, the attachment of the Cpx AH to an SV would promotes the separation of the Cpx AH

from the SNARE bundle and, respectively would stabilize the partially unzippered state of Syb."

## References

[B1] Abderrahmani A, Niederhauser G, Plaisance V, Roehrich M-E, Lenain V, Coppola T, Regazzi R, Waeber G (2004) Complexin I regulates glucose-induced secretion in pancreatic beta-cells. J Cell Sci 117:2239–2247. 10.1242/jcs.01041 15126625

[B2] Alwarawrah M, Wereszczynski J (2017) Investigation of the effect of bilayer composition on PKCα-C2 domain docking using molecular dynamics simulations. J Phys Chem B 121:78–88. 10.1021/acs.jpcb.6b10188 27997184 PMC5582975

[B3] Andreae LC, Burrone J (2018) The role of spontaneous neurotransmission in synapse and circuit development. J Neurosci Res 96:354–359. 10.1002/jnr.24154 29034487 PMC5813191

[B4] Backhaus P, Langenhan T, Neuser K (2016) Effects of transgenic expression of botulinum toxins in *Drosophila*. J Neurogenet 30:22–31. 10.3109/01677063.2016.1166223 27276193

[B5] Brand AH, Perrimon N (1993) Targeted gene expression as a means of altering cell fates and generating dominant phenotypes. Development 118:401–415. 8223268 10.1242/dev.118.2.401

[B6] Buhl LK, Jorquera RA, Akbergenova Y, Huntwork-Rodriguez S, Volfson D, Littleton JT (2013) Differential regulation of evoked and spontaneous neurotransmitter release by C-terminal modifications of complexin. Mol Cell Neurosci 52:161–172. 10.1016/j.mcn.2012.11.009 23159779 PMC3540146

[B7] Bykhovskaia M (2008) Making quantal analysis more convenient, fast, and accurate: user-friendly software QUANTAN. J Neurosci Methods 168:500–513. 10.1016/j.jneumeth.2007.10.006 18045692 PMC2290970

[B8] Bykhovskaia M, Jagota A, Gonzalez A, Vasin A, Littleton JT (2013) Interaction of the complexin accessory helix with the C-terminus of the SNARE complex: molecular-dynamics model of the fusion clamp. Biophys J 105:679–690. 10.1016/j.bpj.2013.06.018 23931316 PMC3736676

[B9] Chang S, Reim K, Pedersen M, Neher E, Brose N, Taschenberger H (2015) Complexin stabilizes newly primed synaptic vesicles and prevents their premature fusion at the mouse calyx of held synapse. J Neurosci 35:8272–8290. 10.1523/JNEUROSCI.4841-14.2015 26019341 PMC6605347

[B10] Chapman ER (2008) How does synaptotagmin trigger neurotransmitter release? Annu Rev Biochem 77:615–641. 10.1146/annurev.biochem.77.062005.101135 18275379

[B11] Chen X, Tomchick DR, Kovrigin E, Araç D, Machius M, Südhof TC, Rizo J (2002) Three-dimensional structure of the complexin/SNARE complex. Neuron 33:397–409. 10.1016/s0896-6273(02)00583-4 11832227

[B12] Cho RW, Kümmel D, Li F, Baguley SW, Coleman J, Rothman JE, Littleton JT (2014) Genetic analysis of the Complexin trans-clamping model for cross-linking SNARE complexes in vivo. Proc Natl Acad Sci USA 111:10317–10322. 10.1073/pnas.1409311111 24982161 PMC4104896

[B13] Fortoul N, Singh P, Hui CY, Bykhovskaia M, Jagota A (2015) Coarse-grained model of SNARE-mediated docking. Biophys J 108:2258–2269. 10.1016/j.bpj.2015.03.053 25954883 PMC4423046

[B14] Fortoul N, Bykhovskaia M, Jagota A (2018) Coarse-grained model for zippering of SNARE from partially assembled states. J Phys Chem B 122:10834–10840. 10.1021/acs.jpcb.8b09502 30408418

[B15] Giraudo CG, Eng WS, Melia TJ, Rothman JE (2006) A clamping mechanism involved in SNARE-dependent exocytosis. Science 313:676–680. 10.1126/science.1129450 16794037

[B16] Giraudo CG, Garcia-Diaz A, Eng WS, Chen Y, Hendrickson WA, Melia TJ, Rothman JE (2009) Alternative zippering as an on-off switch for SNARE-mediated fusion. Science 323:512–516. 10.1126/science.1166500 19164750 PMC3736854

[B17] Hobson RJ, Liu Q, Watanabe S, Jorgensen EM (2011) Complexin maintains vesicles in the primed state in *C. elegans*. Curr Biol 21:106–113. 10.1016/j.cub.2010.12.015 21215631 PMC3048763

[B18] Hua SY, Charlton MP (1999) Activity-dependent changes in partial VAMP complexes during neurotransmitter release. Nat Neurosci 2:1078–1083. 10.1038/16005 10570484

[B19] Hua SY, Raciborska DA, Trimble WS, Charlton MP (1998) Different VAMP/synaptobrevin complexes for spontaneous and evoked transmitter release at the crayfish neuromuscular junction. J Neurophysiol 80:3233–3246. 10.1152/jn.1998.80.6.3233 9862918

[B20] Huntwork S, Littleton JT (2007) A complexin fusion clamp regulates spontaneous neurotransmitter release and synaptic growth. Nat Neurosci 10:1235–1237. 10.1038/nn1980 17873870

[B21] Itakura M, Misawa H, Sekiguchi M, Takahashi S, Takahashi M (1999) Transfection analysis of functional roles of complexin I and II in the exocytosis of two different types of secretory vesicles. Biochem Biophys Res Commun 265:691–696. 10.1006/bbrc.1999.1756 10600482

[B22] Jorquera RA, Huntwork-Rodriguez S, Akbergenova Y, Cho RW, Littleton JT (2012) Complexin controls spontaneous and evoked neurotransmitter release by regulating the timing and properties of synaptotagmin activity. J Neurosci 32:18234–18245. 10.1523/JNEUROSCI.3212-12.2012 23238737 PMC3530744

[B23] Kaeser-Woo YJ, Yang X, Südhof TC (2012) C-terminal complexin sequence is selectively required for clamping and priming but not for Ca2+ triggering of synaptic exocytosis. J Neurosci 32:2877–2885. 10.1523/JNEUROSCI.3360-11.2012 22357870 PMC3742123

[B24] Kavalali ET (2015) The mechanisms and functions of spontaneous neurotransmitter release. Nat Rev Neurosci 16:5–16. 10.1038/nrn3875 25524119

[B25] Krishnakumar SS, Radoff DT, Kümmel D, Giraudo CG, Li F, Khandan L, Baguley SW, Coleman J, Reinisch KM, Pincet F, Rothman JE (2011) A conformational switch in complexin is required for synaptotagmin to trigger synaptic fusion. Nat Struct Mol Biol 18:934–940. 10.1038/nsmb.2103 21785412 PMC3668341

[B26] Krishnakumar SS, Li F, Coleman J, Schauder CM, Kümmel D, Pincet F, Rothman JE, Reinisch KM (2015) Re-visiting the trans insertion model for complexin clamping. Elife 4:e04463. 10.7554/eLife.04463 25831964 PMC4384536

[B27] Kümmel D, Krishnakumar SS, Radoff DT, Li F, Giraudo CG, Pincet F, Rothman JE, Reinisch KM (2011) Complexin cross-links prefusion SNAREs into a zigzag array. Nat Struct Mol Biol 18:927–933. 10.1038/nsmb.2101 21785414 PMC3410656

[B28] Li F, Pincet F, Perez E, Giraudo CG, Tareste D, Rothman JE (2011) Complexin activates and clamps SNAREpins by a common mechanism involving an intermediate energetic state. Nat Struct Mol Biol 18:941–946. 10.1038/nsmb.2102 21785413 PMC3736826

[B29] Link E, Blasi J, Chapman ER, Edelmann L, Baumeister A, Binz T, Yamasaki S, Niemann H, Jahn R (1994) Tetanus and botulinal neurotoxins. Tools to understand exocytosis in neurons. Adv Second Messenger Phosphoprotein Res 29:47–58. 10.1016/s1040-7952(06)80006-6 7848727

[B30] Lippert RA, Predescu C, Ierardi DJ, Mackenzie KM, Eastwood MP, Dror RO, Shaw DE (2013) Accurate and efficient integration for molecular dynamics simulations at constant temperature and pressure. J Chem Phys 139:164106. 10.1063/1.4825247 24182003

[B31] Liu J, Guo T, Wu J, Bai X, Zhou Q, Sui SF (2007) Overexpression of complexin in PC12 cells inhibits exocytosis by preventing SNARE complex recycling. Biochemistry (Mosc) 72:439–444. 10.1134/s0006297907040116 17511609

[B32] Martin JA, Hu Z, Fenz KM, Fernandez J, Dittman JS (2011) Complexin has opposite effects on two modes of synaptic vesicle fusion. Curr Biol 21:97–105. 10.1016/j.cub.2010.12.014 21215634 PMC3026084

[B33] Meinertzhagen IA, Govind CK, Stewart BA, Carter JM, Atwood HL (1998) Regulated spacing of synapses and presynaptic active zones at larval neuromuscular junctions in different genotypes of the flies *Drosophila* and *Sarcophaga*. J Comp Neurol 393:482–492. 10.1002/(SICI)1096-9861(19980420)393:4<482::AID-CNE7>3.0.CO;2-X 9550153

[B34] Mohrmann R, Dhara M, Bruns D (2015) Complexins: small but capable. Cell Mol Life Sci 72:4221–4235. 10.1007/s00018-015-1998-8 26245303 PMC4611016

[B35] Montecucco C, Schiavo G (1993) Tetanus and botulism neurotoxins: a new group of zinc proteases. Trends Biochem Sci 18:324–327. 10.1016/0968-0004(93)90065-u 7901925

[B36] Niemann H, Blasi J, Jahn R (1994) Clostridial neurotoxins: new tools for dissecting exocytosis. Trends Cell Biol 4:179–185. 10.1016/0962-8924(94)90203-8 14731646

[B37] Pellegrini LL, O’Connor V, Lottspeich F, Betz H (1995) Clostridial neurotoxins compromise the stability of a low energy SNARE complex mediating NSF activation of synaptic vesicle fusion. EMBO J 14:4705–4713. 10.1002/j.1460-2075.1995.tb00152.x 7588600 PMC394567

[B38] Phillips JC, Braun R, Wang W, Gumbart J, Tajkhorshid E, Villa E, Chipot C, Skeel RD, Kalé L, Schulten K (2005) Scalable molecular dynamics with NAMD. J Comput Chem 26:1781–1802. 10.1002/jcc.20289 16222654 PMC2486339

[B39] Radoff DT, Dong Y, Snead D, Bai J, Eliezer D, Dittman JS (2014) The accessory helix of complexin functions by stabilizing central helix secondary structure. Elife 3:e04553. 10.7554/eLife.04553 PMC4270070 25383924

[B40] Rizo J, Xu J (2015) The synaptic vesicle release machinery. Annu Rev Biophys 44:339–367. 10.1146/annurev-biophys-060414-034057 26098518

[B41] Rossano AJ, Macleod GT (2007) Loading *Drosophila* nerve terminals with calcium indicators. J Vis Exp. Advance online publication. Retrieved July 30, 2007. doi: 10.3791/250. PMC2557112 18997898

[B42] Sabeva N, Cho RW, Vasin A, Gonzalez A, Littleton JT, Bykhovskaia M (2017) Complexin mutants reveal partial segregation between recycling pathways that drive evoked and spontaneous neurotransmission. J Neurosci 37:383–396. 10.1523/JNEUROSCI.1854-16.2016 28077717 PMC5242395

[B43] Schiavo G, Benfenati F, Poulain B, Rossetto O, Polverino de Laureto P, DasGupta BR, Montecucco C (1992) Tetanus and botulinum-B neurotoxins block neurotransmitter release by proteolytic cleavage of synaptobrevin. Nature 359:832–835. 10.1038/359832a0 1331807

[B44] Shaw DE (2014) Long molecular dynamics simulations of proteins: progress, promise, and problems. Abstr Pap Am Chem S 247.

[B45] Shaw DE, Dror RO, Salmon JK, Grossman JP, Mackenzie KM, Bank JA, Young C, Deneroff MM, Batson B, Bowers KJ, Chow E, Eastwood MP, Ierardi DJ, Klepeis JL, Kuskin JS, Larson RH, Lindorff-Larsen K, Maragakis P, Moraes MA, Piana S, et al. (2009) Millisecond-scale molecular dynamics simulations on Anton. In: Proceedings of the Conference on High Performance Computing, Networking, Storage and Analysis. New York: ACM. 10.1145/1654059.1654126

[B46] Südhof TC (2013) Neurotransmitter release: the last millisecond in the life of a synaptic vesicle. Neuron 80:675–690. 24183019 10.1016/j.neuron.2013.10.022 PMC3866025

[B47] Südhof TC, Rothman JE (2009) Membrane fusion: grappling with SNARE and SM proteins. Science 323:474–477. 10.1126/science.1161748 19164740 PMC3736821

[B48] Trimbuch T, Rosenmund C (2016) Should I stop or should I go? The role of complexin in neurotransmitter release. Nat Rev Neurosci 17:118–125. 10.1038/nrn.2015.16 26806630

[B49] Vaithianathan T, Zanazzi G, Henry D, Akmentin W, Matthews G (2013) Stabilization of spontaneous neurotransmitter release at ribbon synapses by ribbon-specific subtypes of complexin. J Neurosci 33:8216–8226. 10.1523/JNEUROSCI.1280-12.2013 23658160 PMC3694337

[B50] Vaithianathan T, Henry D, Akmentin W, Matthews G (2015) Functional roles of complexin in neurotransmitter release at ribbon synapses of mouse retinal bipolar neurons. J Neurosci 35:4065–4070. 10.1523/JNEUROSCI.2703-14.2015 25740533 PMC4348196

[B51] Vanommeslaeghe K, Hatcher E, Acharya C, Kundu S, Zhong S, Shim J, Darian E, Guvench O, Lopes P, Vorobyov I, Mackerell AD (2010) CHARMM general force field: a force field for drug-like molecules compatible with the CHARMM all-atom additive biological force fields. J Comput Chem 31:671–690. 10.1002/jcc.21367 19575467 PMC2888302

[B52] Vasin A, Bykhovskaia M (2017) Focal macropatch recordings of synaptic currents from the *Drosophila* larval neuromuscular junction. J Vis Exp. Advance online publication. Retrieved September 25, 2017. doi: 10.3791/56493. PMC5752324 28994789

[B53] Vasin A, Volfson D, Littleton JT, Bykhovskaia M (2016) Interaction of the complexin accessory helix with synaptobrevin regulates spontaneous fusion. Biophys J 111:1954–1964. 10.1016/j.bpj.2016.09.017 27806277 PMC5102999

[B54] Wragg RT, Snead D, Dong Y, Ramlall TF, Menon I, Bai J, Eliezer D, Dittman JS (2013) Synaptic vesicles position complexin to block spontaneous fusion. Neuron 77:323–334. 10.1016/j.neuron.2012.11.005 23352168 PMC3559010

[B55] Xu T, Binz T, Niemann H, Neher E (1998) Multiple kinetic components of exocytosis distinguished by neurotoxin sensitivity. Nat Neurosci 1:192–200. 10.1038/642 10195143

[B56] Xue M, Reim K, Chen X, Chao HT, Deng H, Rizo J, Brose N, Rosenmund C (2007) Distinct domains of complexin I differentially regulate neurotransmitter release. Nat Struct Mol Biol 14:949–958. 10.1038/nsmb1292 17828276 PMC4894543

[B57] Yamasaki S, Binz T, Hayashi T, Szabo E, Yamasaki N, Eklund M, Jahn R, Niemann H (1994) Botulinum neurotoxin type G proteolyses the Ala81-Ala82 bond of rat synaptobrevin 2. Biochem Biophys Res Commun 200:829–835. 10.1006/bbrc.1994.1526 7910017

[B58] Yang X, Kaeser-Woo YJ, Pang ZP, Xu W, Südhof TC (2010) Complexin clamps asynchronous release by blocking a secondary Ca(2+) sensor via its accessory α helix. Neuron 68:907–920. 10.1016/j.neuron.2010.11.001 21145004 PMC3050570

[B59] Yang X, Cao P, Südhof TC (2013) Deconstructing complexin function in activating and clamping Ca2+-triggered exocytosis by comparing knockout and knockdown phenotypes. Proc Natl Acad Sci USA 110:20777–20782. 10.1073/pnas.1321367110 24297916 PMC3870694

[B60] Zdanowicz R, Kreutzberger A, Liang B, Kiessling V, Tamm LK, Cafiso DS (2017) Complexin binding to membranes and acceptor t-SNAREs explains its clamping effect on fusion. Biophys J 113:1235–1250. 10.1016/j.bpj.2017.04.002 28456331 PMC5607037

